# Structural and Biochemical Characterization of Poly-ADP-ribose Polymerase from *Trypanosoma brucei*

**DOI:** 10.1038/s41598-017-03751-4

**Published:** 2017-06-16

**Authors:** Teemu Haikarainen, Mariana Schlesinger, Ezeogo Obaji, Silvia H. Fernández Villamil, Lari Lehtiö

**Affiliations:** 1 Biocenter Oulu and Faculty of Biochemistry and Molecular Medicine, University of Oulu, FI-90014 Oulu, Finland; 2 National Institute for Genetic Engineering and Molecular Biology (INGEBI-CONICET), University of Buenos Aires, Buenos Aires, Argentina

## Abstract

*Trypanosoma brucei* is a unicellular parasite responsible for African trypanosomiasis or sleeping sickness. It contains a single PARP enzyme opposed to many higher eukaryotes, which have numerous PARPs. PARPs are responsible for a post-translational modification, ADP-ribosylation, regulating a multitude of cellular events. *T. brucei* PARP, like human PARPs-1-3, is activated by DNA binding and it potentially functions in DNA repair processes. Here we characterized activation requirements, structure and subcellular localization of *T. brucei* PARP. *T. brucei* PARP was found to be selectively activated by 5′ phosphorylated and 3′ phosphorylated DNA breaks. Importantly, the N-terminal region is responsible for high-affinity DNA-binding and required for DNA-dependent enzymatic activation. This module is also required for nuclear localization of the protein in response to oxidative stress. Solution structures of activating and non-activating PARP-DNA complexes were determined with small-angle X-ray scattering revealing distinct differences in their DNA-binding modes.

## Introduction

Poly-APD-ribose polymerases are enzymes catalyzing a covalent addition of ADP-ribose to a target protein, to themselves, or to a growing ADP-ribose polymer. The enzyme family is widespread among eukaryotes, although multicellular eukaryotes contain more family members than lower eukaryotes^1^. In humans the family consists of 17 members, where PARPs are involved in a multitude of cellular processes including DNA damage repair, cell death pathways, and mitosis. Human PARPs (hPARPs)-1-3, whose catalytic activity is induced by DNA-binding^2^ are involved in DNA-damage repair processes^3^. The role of hPARPs-1-3, and especially of hPARP-1, in DNA damage repair is extensively studied and a lot of interest have been devoted to discovery of PARP inhibitors as therapeutics against various forms of cancer^4^.

**Figure 1:**
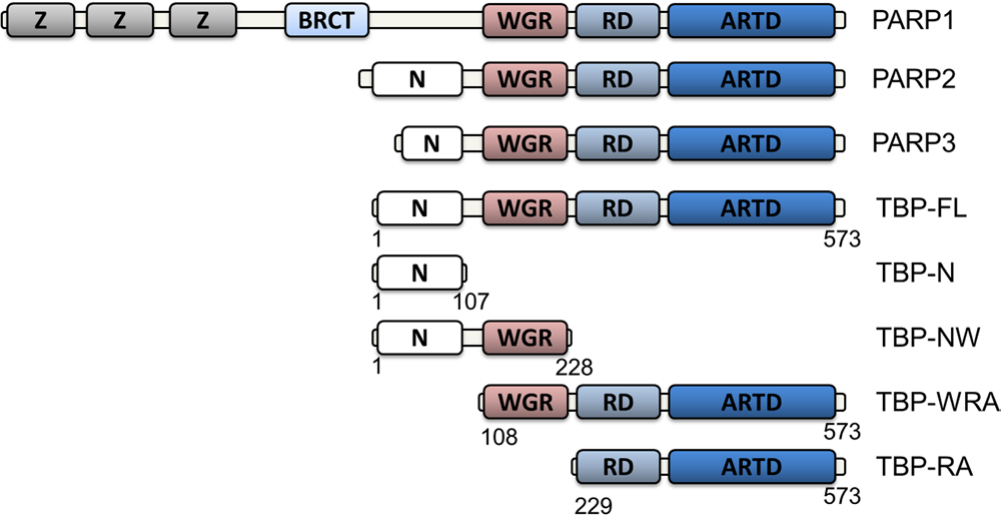
*Tb*PARP constructs. Comparison of T*b*PARP domain structure to human PARPs-2-3 and a schematic presentation of the *Tb*PARP constructs used in the study. Z, zinc finger motif; BRCT, BRCA1 C terminus domain; WGR, WGR domain; RD, regulatory domain; ARTD, catalytic domain; N, N-terminal fragment.

hPARP-1 is a multidomain protein consisting of DNA-binding zinc-fingers, a WGR domain necessary to DNA-dependent activation, a BRCT domain, and regulatory (RD) and catalytic (ARTD) domains responsible for poly-ADP-ribose synthesis (Fig. 1). hPARPs-2 and 3 consist of regulatory and catalytic domains and a WGR domain. Instead of the DNA-binding zinc-fingers, they have N-terminal regions which participate in DNA-binding^2^. hPARPs-2 and 3 were recently shown to prefer specific DNA substrates for activation suggesting their role in specific DNA-repair pathways in opposed to hPARP-1, which is activated by various DNA-damage models^2, 5^.

*Trypanosoma brucei* is a unicellular parasitic protozoa responsible for African trypanosomiasis or sleeping sickness, a scourge in Sub-Saharan Africa for both livestock and human health. Based on sequence comparisons, the parasite contains only a single PARP enzyme (*Tb*PARP), which has a similar domain organization as hPARPs-2 and 3, including the WGR, RD, and ARTD domains (Fig. 1). It also contains a positively charged N-terminus, with higher sequence similarity to hPARP-2 than to hPARP-3.

We have previously shown that *Tb*PARP is enzymatically activated by DNA and migrates to the nucleus after a genomic stress^6^. Interestingly, cytotoxicity induced by hydrogen peroxide was reduced both by down-regulation and inhibition of the catalytic activity of the enzyme suggesting a role for *Tb*PARP in cell death pathways. In addition, inhibition of the catalytic activity of *Tb*PARP did not inhibit the growth of *T. brucei* procyclic and bloodstream forms^6^. These results are in contrast to what have been observed for the related parasite *T. cruzi* ^7^.

Here, in effort to understand the role of PARP in the parasite, we have characterized DNA-binding and DNA-dependent activation properties of the enzyme and especially the role of the N-terminal domain in these processes. We have also studied the role of the N-terminus in the localization of the protein. Finally, we performed structural characterization of *Tb*PARP-DNA-complexes with solution scattering methods. Our results indicate a high degree of substrate selectivity in the DNA activation process and identify the N-terminus as a key domain in DNA-binding, DNA-dependent catalytic activation, and nuclear localization.

## Results

### N-terminus is required for high-affinity DNA binding and activation

We have previously shown that *Tb*PARP is catalytically activated by damaged DNA and it most likely functions in DNA-damage response and DNA-repair like hPARPs-1-3^6^. While hPARP-1 utilizes three zinc-fingers for DNA binding, hPARPs-2 and 3 have a positively charged N-terminus that is utilized for DNA interaction. As the N-terminus of *Tb*PARP is rich in lysines and carries a high positive charge, we hypothesized that the DNA-binding would be mediated by this region.

**Figure 2:**
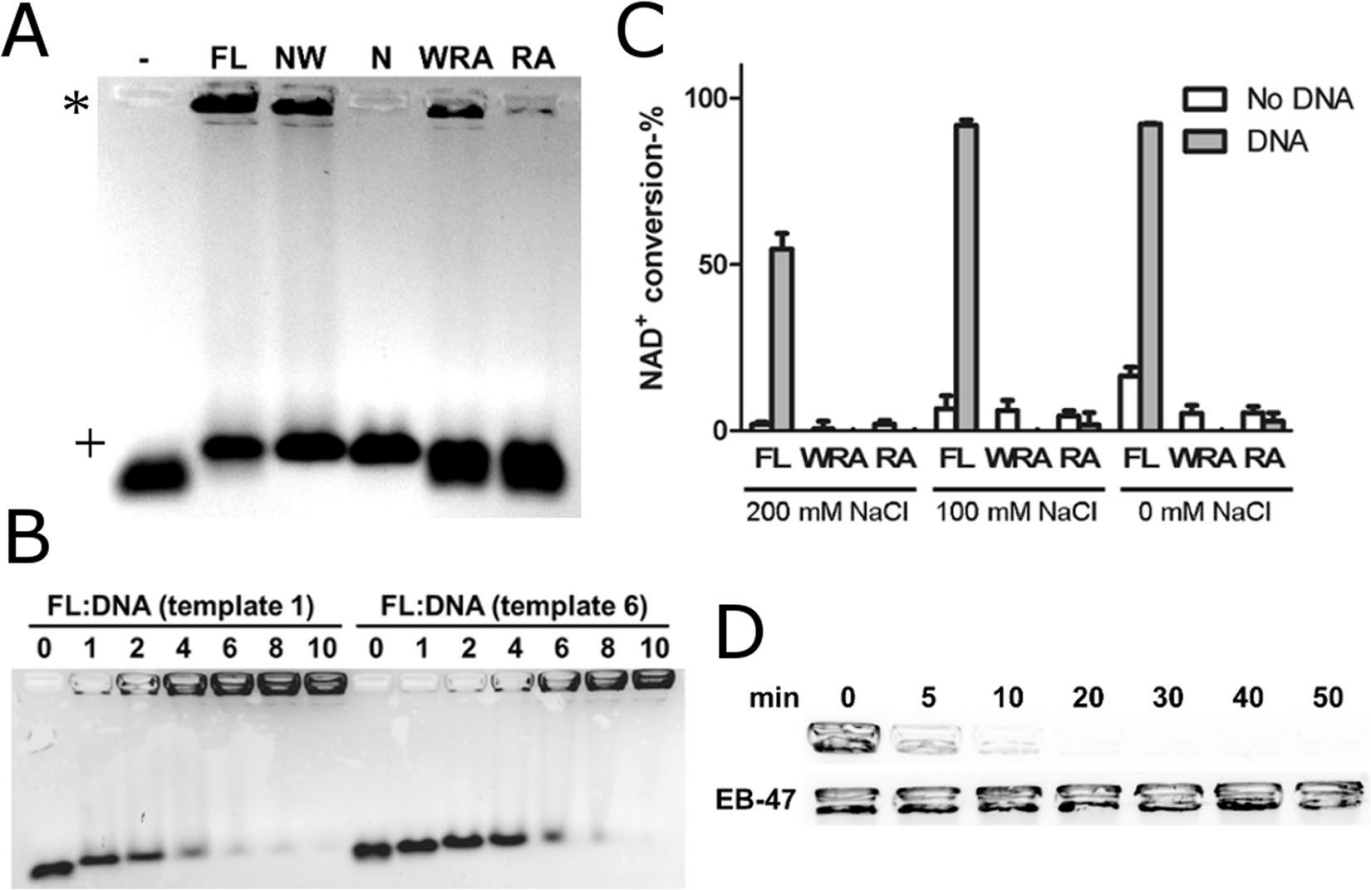
Characterization of DNA-binding properties and DNA-dependent activation of *Tb*PARP. (**A**) The binding of T*b*PARP constructs (2 µM) to DNA template 1 (0.5 µM) were evaluated with agarose gel electrophoresis. (*) Shows the position of the supershifted DNA and (+) displays the smaller DNA shift. (**B**) Concentration-dependent analysis of DNA binding to TBP-FL. TBP-FL was titrated against 0.5 µM DNA (templates 1 and 6) and analyzed with agarose gel electrophoresis. (**C**) DNA dependent activation of TBP-FL, TBP-WRA and TBP-RA using different salt concentrations. Activated DNA was used as a template. (**D**) DNA dissociation from TBP-FL followed by automodification was evaluated as a function of time with agarose gel electrophoresis either without (upper lane) or with (lower lane) inhibitor EB-47 (10 µM).

**Figure 3:**
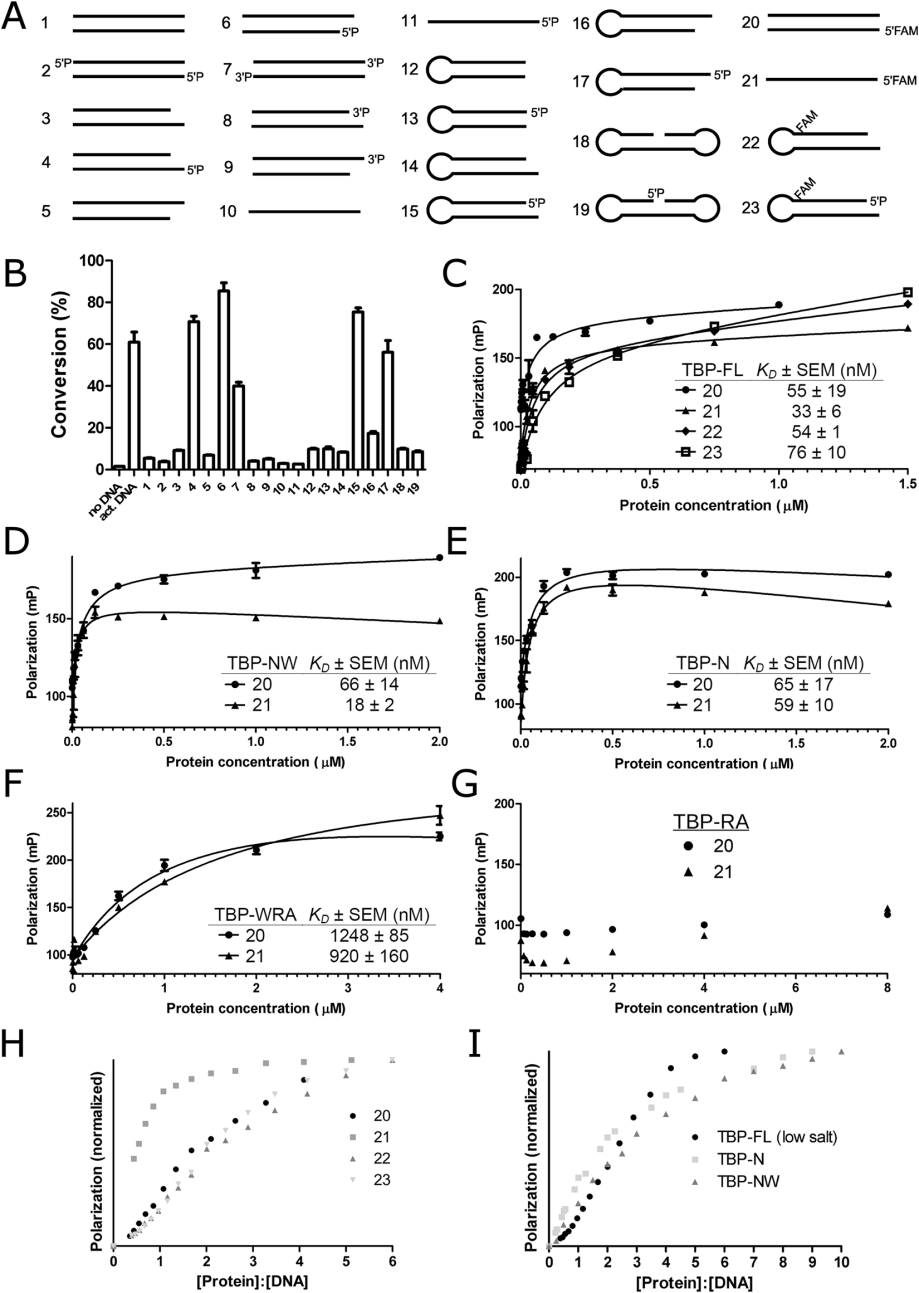
Selectivity, affinity and stoichiometry of DNA binding. (**A**) DNA templates used in activation and fluorescence polarization assays. (**B**) Activation of TBP-FL by DNA templates measured with the fluorescence based activity assay. Activated DNA (act. DNA) was used as a control. (**C**–**G**) DNA-binding affinities for TBP-FL, TBP-NW, TBP-N, TBP-WRA, and TBP-RA were measured with fluorescence polarization assay and are presented in panels C, D, E, F, and G, respectively. A representative curve for each measurement is shown. (**H**) Stoichiometry of TBP-FL binding to oligonucleotides 20, 21, 22, and 23 were measured by fluorescence polarization using saturating DNA concentration (250 nM). 1:1 binding is observed with ssDNA, while 2:1 protein:DNA binding occurs with dsDNA and hairpin DNA. (**I**) Stoichiometry of TBP-FL in low salt (50 mM NaCl) buffer and stoichiometries of TBP-NW and TBP-N constructs using DNA oligonucleotide 20.

We analyzed the DNA-binding properties of the full-length protein and various fragments (Fig. 1) with EMSA using an agarose gel (Fig. 2A). We used double-stranded DNA oligonucleotide (template 1, Fig. 3) in the assay. Full-length protein (TBP-FL) and the fragment containing WGR and N-terminal domains (TBP-NW) resulted in a supershift (DNA retained in the well) of DNA on the gel. The fragment containing N-terminus only (TBP-N) did not retain DNA in the well but still resulted in a shift on the gel compared to the DNA only, indicating binding. The fragment lacking the N-terminus (TBP-WRA) had seemingly lower affinity with DNA as only a minor fraction of the DNA was supershifted on the gel. Therefore, N-terminus was not strictly required for DNA binding but appeared to increase the binding affinity. When both N-terminus and WGR domain were omitted (TBP-RA), the DNA-binding activity was almost completely abolished. With TBP-FL and TBP-NW, two shifts were observed in EMSA: a supershift and a shift corresponding to the position of TBP-N shifted DNA (Fig. 2A). We hypothesized that the smaller shift arises from interactions with the N-terminus, which might be non-specific and have higher on- and off-rate constants. We tested this by titration of the DNA with protein: when protein concentration is increased the DNA gradually shifts towards the well (Fig. 2B). Notably phosphorylated oligonucleotide (template 6, Fig. 2B) seemed to require higher protein concentrations for fully supershifting the DNA indicating higher protein:DNA stoichiometry.

The seemingly lower DNA binding affinity of TBP-WRA prompted us to test whether high affinity binding mediated by the N-terminus was required for DNA-dependent activation. We compared the DNA dependent activation of TBP-FL, TBP-WRA and TBP-RA with a fluorescence-based activity assay measuring NAD^+^ consumption. A robust (≈40-fold) activation of TBP-FL was observed upon incubation with activated DNA (Fig. 2C, 200 mM NaCl). Although TBP-WRA and TBP-RA both retained basal enzymatic activity, there was no increase in enzymatic activity upon incubation with activated DNA. This suggests that the N-terminus is required for both high affinity interaction and DNA-dependent activation of the protein. Since TBP-WRA (and TBP-RA to lower extent) showed interaction with DNA on EMSA at lower salt concentration, we repeated the activity assay at conditions containing 100 mM NaCl and without salt. Higher activation of TBP-FL by activated DNA was observed at lower salt concentrations but these conditions did not result in activation of TBP-WRA or TBP-RA.

hPARPs-1-3 have been shown to be released from DNA upon automodification^2, 8^. This is most likely a result of repulsive interaction between the negatively charged DNA and poly(ADP-ribose) (PAR). We proceeded to test whether the DNA-dependent ADP-ribosylation results in dissociation of the *Tb*PARP-DNA complex. When incubated with NAD^+^, the preformed TBP-FL-DNA complex rapidly dissociates from DNA (Fig. 2D, upper lane). To verify that the automodification is responsible for the dissociation, the preformed complex was incubated in the presence of EB-47, a potent *Tb*PARP inhibitor^6^ before initiating the automodification with NAD^+^. Inhibition of the catalytic activity effectively prevents the dissociation of the complex (Fig. 2D, lower lane).

### *Tb*PARP is selectively activated by DNA

hPARP-1 is activated by various DNA structures whereas hPARPs-2 and 3 appeared to be more selectively activated by DNA^2, 5^. The selectivity of the DNA-dependent activity relates to the biological function of the proteins. Whereas hPARP-1 functions as a DNA damage sensor in the cells, hPARPs-2 and 3 appear to have more specific functions in DNA repair and maintenance of genome integrity^3^. In order to identify an activating DNA oligonucleotide for further studies and to get insight to the function of *Tb*PARP, we tested the activation of the protein by various DNA oligonucleotides (Supplementary Table S1 and Fig. 3A,B). The oligonucleotide panel showed high selectivity in DNA substrate requirement. Non-phosphorylated DNA oligonucleotides showed little to no activation at all. In addition, the phosphorylation of the DNA changed activation properties of certain templates only. Robust activation was specifically seen with 5′ phosphorylated DNA oligonucleotides with 5′ or 3′ overhangs. Both double-stranded and hairpin oligonucleotides with 5′ or 3′ overhangs combined with phosphorylation (templates 4, 6, 15, and 17) resulted in robust activation. Notably 5′ phosphorylation did not change activation properties of blunt end double-stranded DNA (templates 1 and 2), single-stranded DNA (templates 10 and 11), blunt end hairpin DNA (templates 12 and 13) or dumbbell DNA (templates 18 and 19). Oligonucleotides with 3′ phosphorylation behaved differently from 5′ phosphorylated oligonucleotides. No activation was observed with 3′ phosphorylated oligonucleotides containing 5′ or 3′ overhangs (templates 8 and 9). However, 3′ phosphorylated blunt end oligonucleotide (template 7) resulted in moderate catalytic activation.

The DNA-binding affinities were quantified using a fluorescence polarization (FP) assay. The TBP-FL had low nanomolar affinities to dsDNA and ssDNA (templates 20 and 21) and to non-activating and activating hairpin DNA (templates 22 and 23) (Fig. 3C). Importantly, TBP-FL has similar affinities towards both activating and non-activating DNA, implying that binding affinity does not determine enzymatic activation but the protein is capable of identifying specific features on the activating DNA templates. The affinities of the fragments remain essentially unchanged with the omission of the regulatory and catalytic domains (Fig. 3D,E), as could be expected from the results from EMSA. When N-terminus is deleted (TBP-WRA), the affinity drops substantially (Fig. 3F), revealing that while WGR domain is capable of interacting with DNA, N-terminus is required for high affinity interaction. No binding was observed for TBP-RA with FP (Fig. 3G) consistent with EMSA.

Furthermore, we characterized the stoichiometry of the DNA binding. Saturating concentrations of DNA were titrated with protein and analyzed using fluorescence polarization^9, 10^. When using dsDNA, non-activating or activating hairpin templates, an inflection point at 2:1 protein:DNA stoichiometry was observed (Fig. 3H), while ssDNA template displayed a clear 1:1 binding. With templates displaying 2:1 binding, an increasing polarization was observed after the inflection point indicating further, higher order oligomerization or the presence of additional, lower affinity binding sites for DNA in the protein. These interactions could be due to apparent non-specificity of DNA-binding mediated by the N-terminal domain. This was tested by measuring the DNA-binding stoichiometry of TBP-N and TBP-NW fragments. Indeed, these fragments displayed no clear inflection points in the titration indicating adventitious DNA binding (Fig. 3I). Similar non-specific interaction could be seen with TBP-FL when lower salt concentration was used in the assay (Fig. 3I).

### N-terminus is structurally disordered

**Figure 4:**
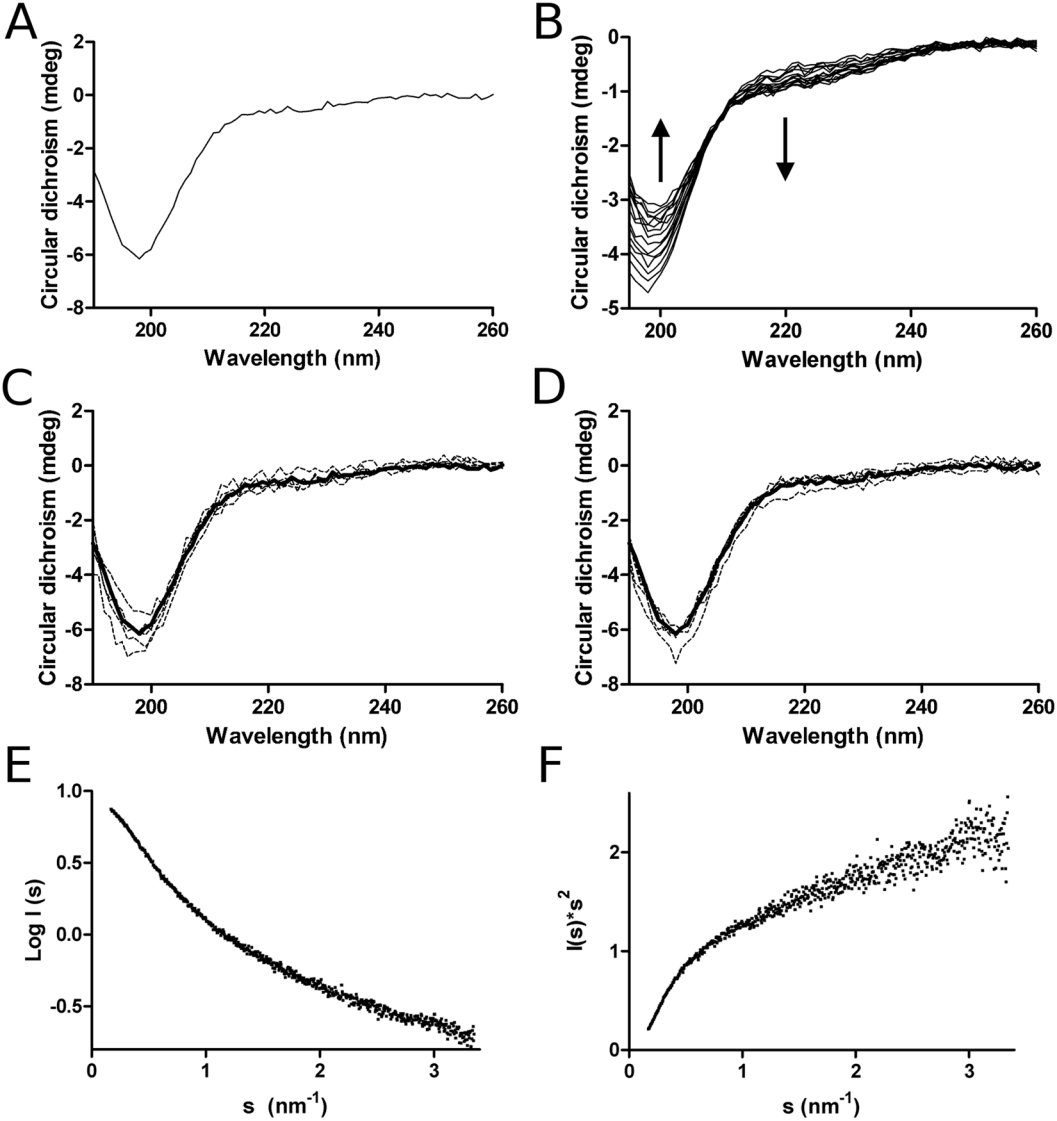
Structural properties of TBP-N. (**A**) CD spectra of TBP-N measured from 190 to 260 nm at 20 °C. (**B**) Thermal denaturation spectra (190–260 nm) of TBP-N from 20 °C to 90 °C. The arrows indicate the spectral shifts upon increasing temperature. (**C**,**D**) Effect of DNA binding to the secondary structure of TBP-N. 21 bp dsDNA (**C**) and 21 bp ssDNA (**D**) was titrated to TBP-N with different molar ratios ranging from 2:1 to 1:16 protein:DNA ratio. Bold line shows the spectra of the native protein and thin lines the DNA titrations. (**E**) SAXS scattering curve of TBP-N. (**F**) Kratky plot of TBP-N showing the disordered state of the domain.

While hPARP-1 interacts with DNA *via* its three zinc-fingers, no domain structure for the DNA binding N-termini of hPARP-2-3 or T*b*PARP have been identified. Many DNA binding proteins harbor a disordered, positively charged terminus responsible for DNA binding^11^. We sought to analyze whether the N-terminus of T*b*PARP would contain regular secondary structure. CD spectra of TBP-N (Fig. 4A) showed features of a disordered polypeptide without significant secondary structure^12^. To verify this observation a thermal denaturation experiment was conducted, which revealed no major changes in the spectra upon heating supporting the disordered nature of the fragment (Fig. 4B). This was further demonstrated with small-angle X-ray scattering (SAXS) analysis: the hyperbolic-like Kratky plot displays the features of a protein having essentially a random coil structure in solution (Fig. 4E,F)^13^.

Some DNA-binding domains undergo transition to a more ordered structure upon binding to DNA^14^. This coupled folding and binding may involve only few residues, or in some cases an entire protein domain. To test whether significant folding of the N-terminus takes place upon DNA binding, the interaction of TBP-N with both dsDNA and ssDNA oligonucleotides was characterized with CD. The spectra of the TBP-N-DNA complexes did not show major changes when compared to the spectra of the protein alone indicating no major folding upon DNA binding (Fig. 4C,D).

### Structural changes upon activation

**Figure 5:**
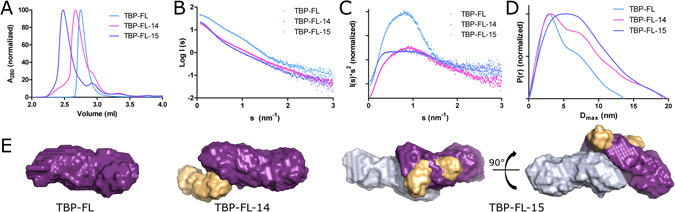
Solution structures of *Tb*PARP and *Tb*PARP-DNA complex. HPLC chromatograms (**A**), SAXS scattering curves (**B**), Kratky plots (**C**), P(r) functions (**D**) and *ab initio* reconstructions of TBP-FL and TBP-FL-DNA complexes with DNA templates 14 and 15 (**E**). The DNA templates in the complexes are colored yellow, and the two *Tb*PARP monomers are colored in purple and grey in the *ab initio* model of TBP-FL-15 complex.

The observation that DNA binding induces protein oligomerization prompted us to study the process in more detail with SAXS (Fig. 5). We noticed that the complexes of TBP-FL with non-activating and activating DNA (templates 14 and 15, the difference of a 5′ phosphate) elute with different retention volumes, indicating significantly higher particle size for the latter (Fig. 5A). Unlike the stoichiometries from fluorescence polarization, the protein-DNA complexes eluted as single peaks from the HPLC, indicating that at these conditions stable oligomers are formed. We proceeded to characterize the complexes with SAXS. We first characterized TBP-FL and found that the monomeric enzyme is characterized by an Rg of 4.04 nm with maximum dimension of 13.6 nm (Tables 1 and 2). The molecular mass derived from the SAXS data (63 and 71 kDa), is very close to the calculated monomeric weight of the protein (65 kDa). The elevated baseline at high s in the Kratky plot and the extended conformation derived from the pair-distance distribution (P(r)) function could arise from the disordered regions in the protein and multiple domains, respectively (Fig. 5C,D).

**Table 1:** Summary of SAXS analysis.

Protein	D_max_ (nm)	Porod volume (nm^3^)	MW_exp_ (kDa)	MW_calc_ *ab initio* (kDa)	MW_calc_ Porod (kDa)	NSD ± std
TBP-N	12.3	25	11	18	15	0.703 ± 0.028
TBP-FL	13.6	106	65	71	63	0.631 ± 0.049
TBP-FL-14	19.3	120	85	86	71	0.665 ± 0.030
TBP-FL-15	19.8	266	150	131	156	0.802 ± 0.026

MW_exp_ = expected molecular weight, MW_calc_ Porod = calculated molecular weight obtained by dividing the Porod volume by 1.7, MW_calc_ *ab initio* = calculated molecular weight obtained from DAMAVER, NSD = normalized spatial discrepancy for *ab initio* modelling.

**Table 2:** SAXS data processing.

Protein	I(0)	R_g_ from Guinier analysis (nm)	R_g_ from GNOM analysis (nm)
TBP-N	8.49	3.46	3.67
*4.8* *mg/ml*	8.57	3.49	3.73
*7.1* *mg/ml*	8.28	3.57	3.71
TBP-FL	4.60	4.04	4.74
*2.4* *mg/ml*	4.59	4.11	4.12
*3.5* *mg/ml*	4.56	4.01	4.13
TBP-FL-14	19.77	5.45	5.65
*9.2* *mg/ml*	18.59	5.52	5.72
*13.8* *mg/ml*	20.39	5.76	5.99
TBP-FL-15	23.82	5.78	5.89
*6.0* *mg/ml*	22.00	5.89	5.95
*9.0* *mg/ml*	24.30	6.06	6.12

Forward scattering (I(0)) and radius of gyration (R_g_) is shown for both merged data and data for low and high concentrations (in italics). I(0) values have been normalized to concentration.

TBP-FL in complex with non-activating DNA has a molecular mass consistent with a 1:1 complex (Table 1). The maximum particle dimension (D_max_) is highly increased compared to TBP-FL indicating a flexible, non-compact particle. A two-phase *ab initio* reconstruction of the complex indicates that the binding of DNA takes place at one end of the protein molecule, likely at the N-terminus, with little to no interaction with the rest of the protein. The bound DNA extends outward from the protein leading to an elongated particle resulting in increased flexibility as seen in the Kratky plot (Fig. 5C) and extended D_max_ (Fig. 5D).

When binding to activating DNA, the molecular mass of the complex suggests 2:1 protein:DNA ratio (Table 1) agreeing with the fluorescence polarization results (Fig. 3H) and EMSA titrations (Fig. 2B) which suggested a higher protein:DNA stoichiometry with activating DNA. The maximum particle dimension (D_max_) is highly increased compared to TBP-FL in agreement with a larger particle size. The shape of the P(r) function indicates compaction of the particle in comparison to the TBP-FL (Fig. 5D). The two-phase *ab initio* reconstruction of the complex resulted in a TBP-FL dimer with DNA bound between the two protein monomers (Fig. 5E). The majority of the DNA appears to be complexed with one protein monomer, while the other monomer seems to mainly interact with another protein monomer. This implies the formation of a non-symmetrical complex, where the second monomer might not get activated by DNA-binding.

### N-terminus is required for nuclear localization

**Figure 6:**
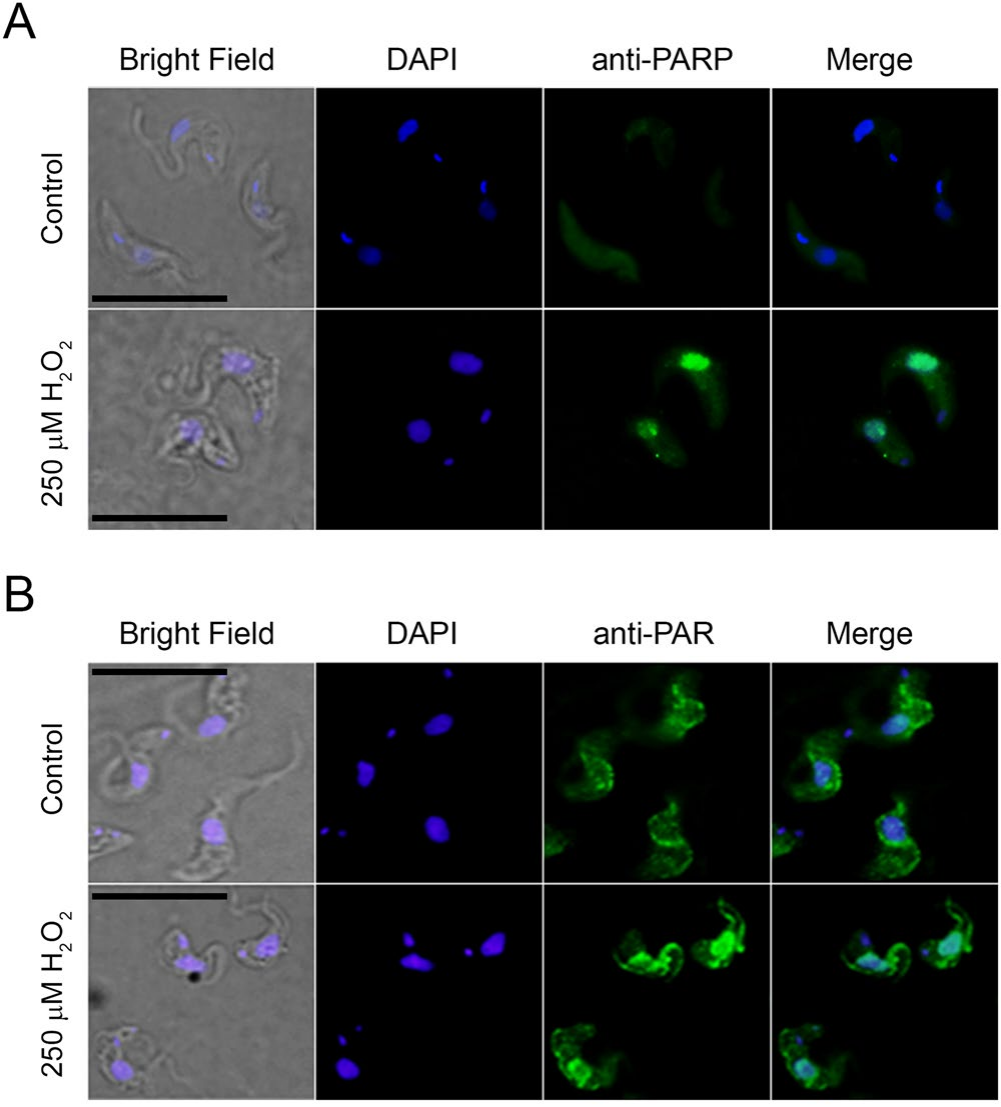
*Tb*PARP (**A**) and PAR (**B**) localization was detected with anti-*Tb*PARP and anti-PAR polyclonal antibodies, respectively, in wild type bloodstream parasites. DAPI was used to identify nuclear and kinetoplastid DNA. Merge panels show that both *Tb*PARP and PAR are localized to the nucleus upon hydrogen peroxide treatment. Bar: 5 µm.

**Figure 7:**
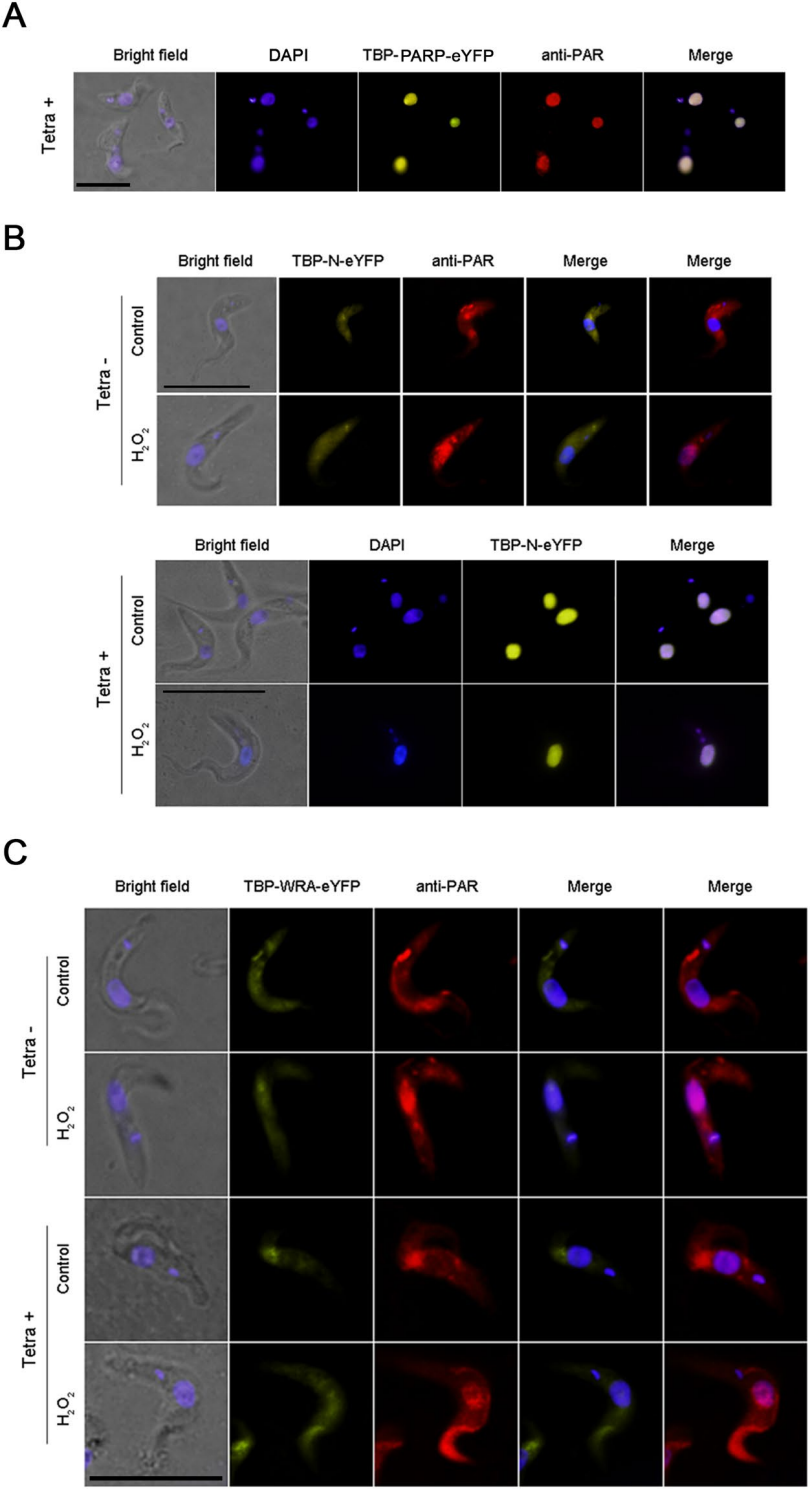
Localization of fusion proteins in transgenic procyclic parasites overexpressing full-length protein (TBP-FL-eYFP) (**A**); or only the N-terminus (TBP-N-eYFP) (**B**) or the protein without the N-terminus (TBP-WRA-eYFP) (**C**) subjected to hydrogen peroxide (H_2_O_2_) treatment. PAR localization was also revealed with anti-PAR polyclonal antibody. DAPI was used to identify nuclear and kinetoplastid DNA. The protein overexpression was induced by tetracycline. The merge panels show localization of TBP-FL-eYFP and TBP-N-eYFP to the nucleus irrespective of hydrogen peroxide treatment, while TBP-WRA-eYFP is not localized to the nucleus even after treatment with hydrogen peroxide. Bar: 5 µm.

We have previously shown that *Tb*PARP is localized to the nucleus in response to oxidative stress in procyclic parasites^6^. Here the nuclear localization of *Tb*PARP was investigated in the bloodstream form of the parasite with indirect immunofluorescence microscopy. Similar to procyclic form of the parasite, *Tb*PARP was found mainly in the cytoplasm, but after treatment with H_2_O_2_ it was localized in the nucleus (Fig. 6A). Also PAR was found to be localized in the cytoplasm in untreated cells, and strong induction in its production was seen in the nucleus after the H_2_O_2_ treatment (Fig. 6B). On the other hand, procyclic transgenic lines which over-expressed the fusion protein *Tb*PARP-eYFP presented the heterologous enzyme in the nucleus, even in absence of a genotoxic insult (Fig. 7A). To further characterize the nuclear localization we proceeded to test whether the N-terminus is required for the nuclear import. Proteins that are transported to nucleus *via* the importin-α/β complex are recognized by their nuclear localization signals (NLS) by importin α^15^. NLS typically consists of short stretches of lysines and arginines making the T*b*PARP N-terminus a good candidate for the location of the NLS.

To test the role of the N-terminus in protein localization we used procyclic parasites expressing the TBP-N-eYFP and TBP-WRA-eYFP constructs under the control of tetracyclin inducible promoter. TBP-N-eYFP fusion protein appeared in the nucleus in 2-day tetracycline induced procyclic parasites, even in absence of H_2_O_2_ treatment, unlike the uninduced control parasites (Fig. 7B). Notably, the TBP-WRA is localized in the cytoplasm without the H_2_O_2_ treatment and does not appear in the nucleus even after H_2_O_2_- induced damage (Fig. 7C). Together the localization of the eYFP constructs suggest that the N-terminus is responsible for the nuclear localization of T*b*PARP.

## Discussion

Unicellular protozoan parasite, *T. brucei* has only one PARP^16^, which is activated by DNA^6^. This is in agreement with PARPs in other trypanosomatids^16, 17^. The characterized DNA-dependent PARPs function in DNA repair pathways, and have been found to be essential in mice, where PARP-1/2 knockout is embryonically lethal^18^. Previously, we found that *T. cruzi* amastigotes are sensitive to PARP inhibition^7^. In contrast, *Tb*PARP is not necessary for the viability of *T. brucei* ^6, 19^ indicating differences in the roles of PARPs between the parasites.

Although *Tb*PARP is able to bind various DNA substrates it requires specifically DNA templates with single-strand overhangs combined with 5′ phosphorylation for robust activation. Activation was also observed with 3′ phosphorylation, but only when present at a blunt end of the DNA. Notably, the binding affinities do not change when binding to activating or non-activating DNA, implying the DNA structure does not affect the binding process. Same behavior has been observed also for human DNA-dependent PARPs^2^. N-terminus acts as a central module for both DNA-binding and DNA-dependent catalytic activation. This is in contrast to hPARPs-2-3, in which N-terminus is not necessary for DNA-dependent activation^2, 5^. It seems that the N-terminus functions as a non-selective affinity enhancer in the binding event and the activation is determined by specific features of the DNA template interacting, possibly, with the WGR domain.

Structurally disordered, positively charged tails are common in DNA-binding proteins and are often utilized as affinity tuners in protein-DNA-interactions. CD and SAXS analysis revealed that N-terminus of *Tb*PARP is structurally disordered. Disordered N-terminus has also recently been observed for hPARP-2^5, 20^. Importantly, no folding of the N-terminus was observed in response to DNA binding. This further supports the non-selective DNA-binding mode of the N-terminus. Accordingly, the selectivity for the DNA-dependent activation should arise from the WGR domain, the other domain capable of DNA binding. Indeed, WGR domain has been found essential for the DNA-dependent activity of hPARPs-2-3^2^.

Despite extensive studies on hPARPs-1-3, it is not clear whether the enzymes interact with DNA as monomers or dimers. hPARP-1 does not require specific DNA templates for activation and it has been found to form 1:1 complexes with single-strand break and blunt-end DNA^21, 22^, and 2:1 complexes with DNA overhangs^23, 24^. We studied the stoichiometry *Tb*PARP-DNA interaction with FP, and found that the protein interacts with ssDNA as a monomer but either oligomerizes upon binding to dsDNA or has additional lower affinity binding sites for dsDNA. Protein oligomerization or the presence of multiple DNA binding sites was observed when binding to both activating and non-activating DNA templates. However in EMSA (Fig. 2B) higher protein concentrations were required for complexation of activating DNA than non-activating DNA, indicating oligomerization of the protein when binding to activating DNA. When the protein-DNA complexes were separated by size-exclusion chromatography, stable complexes formed unlike in FP measurements. This is likely due to different conditions, *e.g*. higher salt concentration used in SEC. The purified complexes where analyzed by solution scattering methods, which indicated dimerization of the protein upon binding to activating DNA, while non-activating DNA bound to the protein with 1:1 stoichiometry. In the two-phase *ab initio* model two protein molecules were complexed with one activating DNA molecule. The DNA is primarily complexed to one PARP monomer, and it extends also outside the N-terminus, interacting possibly with the WGR domain. The second protein monomer seems to be mainly complexed with the other protein and not significantly interacting with DNA. In this case, the binding of additional protein monomers could be mediated by protein-protein –interactions between the PARPs rather than with the DNA, as suggested by the binding of the second monomer in the SAXS model (Fig. 5).

*Tb*PARP is localized mostly in the cytosol but upon genotoxic stimulus it moves to the nucleus. N-terminus is required for this process as its deletion abolishes nuclear localization. In addition, N-terminus alone is directed to nucleus even in the absence of genotoxic stimulus. No canonical nuclear localization signal has been identified in *T. brucei* but the N-terminus contains several lysine clusters that may be used for nuclear localization. According to nuclear localization upon DNA damage (oxidative stress) *Tb*PARP appears to have a role in DNA repair processes. Based on the specific DNA template requirement for activation, it likely is involved in certain repair pathways like hPARP-2-3 and does not function as a global DNA repair protein like hPARP-1. Therefore the parasite seems to lack a PARP-1-like DNA damage sensor. The indication that *Tb*PARP is not essential for the parasite, even in the presence of genotoxic stimulus^6^ indicates it does not participate in DNA double-strand break (DSB) repair through homologous recombination (HR) pathway. As nonhomologous end joining (NHEJ) is absent in trypanosomatids, DSBs are solely repaired through HR. Microhomology-mediated end joining (MMEJ) is usually viewed as a backup mechanism for NHEJ but in *T. brucei* it dominates end joining. However, little is known about this repair pathway in *T. brucei* ^25^. This raises the possibility of the role of *Tb*PARP in MMEJ pathway as supported by the strict DNA template requirement for enzymatic activation but this remains to be addressed in future studies.

## Methods

### Molecular cloning and DNA oligonucleotides

*Tb*PARP constructs (GenBank:DQ679800) TBP-RA and TBP-N (Fig. 1) were cloned by PCR extension cloning to pNIC28-Bsa4 vector (N-terminal 6xHis-tag with a TEV-protease cleavage site) and TBP-FL, TBP-WRA and TBP-NW constructs were cloned by PCR extension cloning to pNH-trxt vector with an N-terminal 6xHis-tag and thioredoxin tag with a TEV-protease cleavage site. Activated DNA was prepared by incubating calf thymus DNA (Sigma) with DnaseI^26^. All DNA oligonucleotides were purchased from IDT (Integrated DNA Technologies, Inc.).

### Protein expression

Proteins were expressed in *E. coli* Rosetta 2 (DE3) cells grown in Terrific broth auto-induction media (Formedium) supplemented with 8 g/l glycerol, 50 µg/ml of kanamycin and 34 µg/ml of chloramphenicol. The cells were grown at 37 °C until OD_600_ reached 1.0 and the temperature was lowered to 20 °C for protein expression. After 20 hours the cells were collected, suspended in lysis buffer (50 mM HEPES pH 7.4, 0.5 M NaCl, 10% glycerol, 10 mM imidazole 0.5 mM TCEP) and stored at −20 °C.

### Protein purification

Cell suspension was supplemented with 0.5 mg/ml lysozyme (Sigma-Aldrich), 20 µg/ml DNaseI (Roche) and 0.1 mM Pefabloc SC (Sigma-Aldrich). Cells were lysed by sonication and the cell debris was cleared by centrifugation (31000 × *g*, 45 min at 4 °C). Supernatant was filtered through a 0.45 µm filter and loaded on a 1 ml HisTrap Chelating HP column (GE Healthcare) charged with Ni^2+^. The column was subsequently washed with lysis buffer, wash buffer 1 (50 mM HEPES pH 7.4, 0.5 M NaCl, 10% glycerol, 25 mM imidazole, 0.5 mM TCEP) and wash buffer 2 (50 mM HEPES pH 7.4, 1 M NaCl, 10% glycerol, 10 mM imidazole, 0.5 mM TCEP). Protein was eluted by elution buffer (50 mM HEPES pH 7.4, 500 mM NaCl, 10% glycerol, 500 mM imidazole, 0.5 mM TCEP).

For TBP-FL, TBP-NW and TBP-N the NaCl concentration of the buffer was diluted to 300 mM with 20 mM HEPES pH 7.0, 10% glycerol, 0.5 mM TCEP. The protein solution was loaded on to a 5 ml HiTrap Heparin HP column (GE Healthcare) and the column was washed with 20 mM HEPES pH 7.0, 300 mM NaCl, 10% glycerol, 0.5 mM TCEP. The protein was gradient eluted against 20 mM HEPES pH 7.0, 1 M NaCl, 10% glycerol, 0.5 mM TCEP.

For TBP-WRA the NaCl concentration was diluted to 100 mM with 0.1 M Tris pH 8.5, 10% glycerol, 0.5 mM TCEP and pH of the solution was adjusted to 8.5 with NaOH. The protein was loaded on to a 1 ml Q HP column (GE Healthcare) and the column was washed with 20 mM Tris pH 8.5, 100 mM NaCl, 10% glycerol, 0.5 mM TCEP. The protein was gradient eluted against 20 mM Tris pH 8.5, 1 M NaCl, 10% glycerol, 0.5 mM TCEP.

The fusion-tags were cleaved with TEV-protease at 4 °C overnight^27^. The solutions were loaded again on a Ni^2+^ charged 1 ml HisTrap Chelating HP column and the proteins were collected from the flowthrough.

For TBP-WRA the NaCl concentration was diluted to 100 mM with 50 mM HEPES pH 7.0, 10% glycerol, 0.5 mM TCEP. The protein was loaded on to a 1 ml SP HP column (GE Healthcare) and washed with 50 mM HEPES pH 7.0, 100 mM NaCl, 10% glycerol, 0.5 mM TCEP. The protein was gradient eluted against 50 mM HEPES pH 7.0, 1 M NaCl, 10% glycerol, 0.5 mM TCEP.

The proteins were further purified with HiLoad 16/600 Superdex 75 (TBP-N, TBP-NW and TBP-RA) or HiLoad 16/600 Superdex 200 (TBP-FL and TBP-WRA) pre-equilibrated with gel filtration buffer (20 mM HEPES pH 7.4, 0.5 M NaCl, 10% glycerol, 0.5 mM TCEP). The purified proteins were analyzed with SDS-PAGE (Supplementary Fig. S1), flash-frozen in liquid N_2_ and stored at −70 °C.

### Activity assay

Assay was conducted as reported earlier^6, 7^. Briefly, the enzymatic reactions were carried on a 96-well plate in quadruplicates. After the enzymatic reaction 20 µl of 20% acetophenone in ethanol and 20 µl of 2 M KOH were added to the plate and after 10 minutes of incubation 90 µl of formic acid was added. The fluorescence intensity was measured after 20 minutes using Tecan Infinity M1000 with excitation/emission wavelengths of 372 nm and 444 nm, respectively. Buffer in all the assays was 0.1 M Na_2_HPO_4_/NaH_2_PO_4_ pH 7.2, 0.5 mM TCEP, 0.5 mg/ml BSA, 200 mM NaCl. The concentration of activated DNA (calf thymus DNA treated with DNase)^26^ in the assay was 10 µg/ml and concentration of the oligonucleotides was 2.5 µM.

### Electrophoretic mobility shift and automodification assays

For electrophoretic mobility shift assay (EMSA) 0.5 µM DNA was incubated with 2 µM protein in 50 mM Tris pH 7, 0.1 mM TCEP, 0.1 mM EDTA, 5% glycerol, 100 mM NaCl at room temperature for 1 hour. The automodification assay was performed by incubating 2 µM TBP-FL with 500 nM DNA in 0.1 M Na_2_HPO_4_/NaH_2_PO_4_ pH 7.2, 0.5 mM TCEP, 0.5 mg/ml BSA, 200 mM NaCl with and without 10 µM EB-47 at room temperature for 1 hour. Automodification reaction was initiated by the addition of 100 µM NAD^+^ at different time points. EMSA samples were resolved on 0.5% agarose gel with 1xTBE buffer at 4 °C at 80 V for 80 minutes and automodification assay samples were analyzed on 0.8% agarose gel with 1xTBE buffer at 4 °C at 80 V for 1 h. The gels were stained with 1× GelRed (Biotium) or Midori Green Direct (Nippon Genetics) for automodification assay and EMSA, respectively.

### Fluorescence polarization assay

Fluorescence polarization was measured in a 96-well U-bottom black propylene plates (Greiner BioOne) in a final volume of 180 µl. The reaction was carried out in 10 mM HEPES pH 8, 0.1 mM TCEP, 0.1 mM EDTA, 175 mM NaCl and contained 5 nM of labelled DNA and various concentrations of protein. In the case of stoichiometric titrations, saturating (250 nM) concentration of labelled DNA was used. The plate was incubated at 25 °C with shaking at 300 rpm using PST-60 HL plus Thermo Shaker (Biosan, Riga, Latvia) for 80 min. Fluorescence polarization was measured on a Tecan Infinity M1000 with excitation/emission wavelengths of 475 and 520 nm, respectively. Three independent measurements (two with TBP-WRA) were fitted separately with a one-site binding model using GraphPad Prism version 5.04 for Windows (GraphPad Software).

### Circular dichroism spectroscopy

Circular dichroism (CD) spectra of TBP-N were recorded with Chirascan™ CD Spectrometer (Applied Photophysics Ltd) using a wavelength range from 190–260 nm. Measurements were performed at 20 °C in a 1 mm-path length quartz cuvette in 10 mM NaH_2_PO_4_/Na_2_HPO_4_ pH 7.2, 150 mM (NH_4_)_2_SO_4_. The data were analyzed using Pro-Data™ Software Suite (Applied Photophysics Ltd). Thermal denaturation spectra were measured from 20 °C to 90 °C for every 5 °C. DNA titrations were performed from 2:1 to 1:16 protein:DNA molar ratios.

### Small-angle X-ray scattering

Prior to measurements, the protein-DNA-complexes were purified with size-exclusion chromatography using KW403-4F HPLC column (Shodex) in 20 mM HEPES pH 7.0, 300 mM NaCl, 0.5 mM TCEP. The data were collected on BM29 beamline at ESRF (Grenoble, France) using protein concentrations between 1–9 mg/ml. The data were analyzed using the ATSAS suite^28^ (Table 2). Data processing and R_g_ determination were done with PRIMUS^29^. The Guinier plots were displayed with ScÅtter (Supplementary Fig. S2) (www.bioisis.net). Distance distribution functions (Pr) and maximum distances (D_max_) were determined with GNOM^30^ and Porod volumes were determined with DATPOROD^28^. Molecular weight estimate was obtained by dividing the Porod volume by 1.7. *Ab initio* model of the TBP-FL was generated with DAMMIF^31^ and 20 individual models were averaged with DAMAVER^32^. *Ab initio* models of the DNA complexes were created with MONSA^33^. 10 independent MONSA modellings were performed and averaged using DAMAVER. The theoretical scattering curves for DNA used in MONSA modelling was generated with CRYSOL^34^.

### Parasite cultures

Cells of the procyclic form of *T. brucei* strain 29–13^35^ were cultured at 28 °C in SDM-79 (Bioscience) supplemented with 10% (v/v) FCS and 0,002% hemin. Cells of the bloodstream form of *T. brucei* strain 427 90–13^35^ were cultured at 37 °C in HMI-11 (Iscore’s Modified Dulbecco’s Medium (Invitrogen)), 100 mg/l sodium pyruvate, 136.1 mg/l hypoxantine, 38.7 mg/l thymidine, 28.22 mg/l bathocuproinedisulfonic acid, 181.8 mg/l L-cysteine, 3.024 mg/l sodium carbonate, 196 µM β-mercaptoethanol supplemented with 10% (v/v) FCS.

### Procyclic transgenic lines

TBP-N and TBP-WRA were sub-cloned into the expression vector p2216^36^ to overexpress TBP-N-eYFP and TBP-WRA-eYFP fusion genes, respectively. The constructs were linearized with NotI and transfected into procyclic *T. brucei* strain 29–13 by electroporation. Briefly, 10^9^ cells were harvested, washed once with cytomix buffer (120 mM KCl, 0.15 mM CaCl_2_, 10 mM K_2_HPO_4_, 25 mM HEPES (pH 7.6), 2 mM EDTA, 5 mM MgCl_2_), and suspended in 0.5 ml of the same buffer containing 10 μg of the construct. Electroporation was carried out in a 2-mm cuvette using the Gene Pulser (Bio-Rad) with parameters set as follows: 1.5 kV voltage, 25 microfarads capacitance and 2 pulses. The cells were transferred to 10 ml of antibiotics-supplemented SDM-79 medium immediately after electroporation and incubated at 28 °C for 24 h. The transfectants were then selected under 20 μg/ml zeocin until stable cell lines were grown up after about 3 weeks of continuous incubation. For induction of overexpression of fusion proteins, cells were cultured in the above medium containing 1.0 μg/ml tetracycline. The presence of the over-expressing transgenes was confirmed by Southern-blot and protein expression was determined by Western blot analysis.

### Immunolocalization assays

Wild type bloodstream cultures were grown in HMI-11 medium for 24 h up to a density of 5 × 10^5^ parasites/ml, and treated with 250 µM of H_2_O_2_ for 10 minutes in the same culture media. Transgenic procyclic cultures were grown in SDM-79 medium up to a density of 5 × 10^6^ parasites/ml and treated with 500 µM of H_2_O_2_ for 10 minutes.

Parasites were fixed with 3.8% (W/V) formaldehyde in PBS at 4 °C, permeabilized with fresh PBS-0.1% Triton X-100 and blocked at room temperature for 1 h. PAR was detected with 1:500 rabbit polyclonal anti- PAR antibody (BD) followed by 1:500 Alexa Fluor 488 goat anti-rabbit IgG antibody (Invitrogen). *Tb*PARP was detected with 1:100 rabbit polyclonal anti-*Tb*PARP antibody (GenScript), followed by 1:500 Alexa Fluor 594 goat anti-rabbit IgG antibody (Invitrogen). Fusion proteins TBP-PARP-eYFP, TBP-N-eYFP and TBP-WRA-eYFP were localized with fluorescence of eYFP. Excess of antibody was removed by 3 × 5 min washes in PBS, and nuclear and kinetoplast DNA stained with 2 μg/ml DAPI (Sigma). Coverslips were washed with distilled water and mounted in Mowiol and then visualized using an Olympus BX41 microscope.

## Electronic supplementary material

Supplementary file
