# Supplementary material for: Structural and Biochemical Characterization of Poly-ADP-ribose Polymerase from Trypanosoma brucei
Source: Sci Rep. 2017 Jun 16;7:3642. doi: 10.1038/s41598-017-03751-4 (PMC5473844; doi:10.1038/s41598-017-03751-4)
Supplement: Supplementary file 1 — Supplementary file [file 41598_2017_3751_MOESM1_ESM.pdf]

Supplementary Information

Structural and Biochemical Characterization of Poly-ADP-ribose Polymerase from *Trypanosoma brucei*

Teemu Haikarainen[1], Mariana Schlesinger[2], Ezeogo Obaji[1], Silvia H. Fernández Villamil[2] & Lari Lehtiö[1,*]

Content:

Supplementary Table S1

Supplementary Figure S1

Supplementary Figure S2

Supplementary Table S1. DNA templates and sequences.

DNA template	Sequence	Notes
1	5' CCAGGACCAGGGCGCAGATCACCTTGTTCTCCA 3'	
	3' GGTCCTGGTCCCGCGTCTAGTGGAACAAGAGGT 5'	
2	5' CCAGGACCAGGGCGCAGATCACCTTGTTCTCCA 3'	with 5' phosphate
	3' GGTCCTGGTCCCGCGTCTAGTGGAACAAGAGGT 5'	with 5' phosphate
3	5' GCTCTAGTGTAGCATGAGCCTTAGCGCG 3'	
	3' CGAGATCACATCGTACTCGGAATCGCGCTCGA 5'	
4	5' GCTCTAGTGTAGCATGAGCCTTAGCGCG 3'	
	3' CGAGATCACATCGTACTCGGAATCGCGCTCGA 5'	with 5' phosphate
5	5' CGCGCTAAGGCTCATGCTACACTAGAGCTCGA 3'	
	3' GCGCGATTCCGAGTACGATGTGATCTCG 5'	
6	5' CGCGCTAAGGCTCATGCTACACTAGAGCTCGA 3'	
	3' GCGCGATTCCGAGTACGATGTGATCTCG 5'	with 5' phosphate
7	5' CCAGGACCAGGGCGCAGATCACCTTGTTCTCCA 3'	with 3' phosphate
	3' GGTCCTGGTCCCGCGTCTAGTGGAACAAGAGGT 5'	with 3' phosphate
8	5' GCTCTAGTGTAGCATGAGCCTTAGCGCG 3'	with 3' phosphate
	3' CGAGATCACATCGTACTCGGAATCGCGCTCGA 5'	with 3' phosphate
9	5' CGCGCTAAGGCTCATGCTACACTAGAGCTCGA 3'	with 3' phosphate
	3' GCGCGATTCCGAGTACGATGTGATCTCG 5'	
10	5' TTTTTTTTTTTTTTTTTTTTTTTTTTTTTT 3'	
11	5' TTTTTTTTTTTTTTTTTTTTTTTTTTTTTT 3'	with 5' phosphate
12	5' GCTCTAGTGTAGCATGAGCCTTAGCGCGTTTTCGCGCTAAGGCTCATGCTACACTAGAGC 3'	hairpin
13	5' GCTCTAGTGTAGCATGAGCCTTAGCGCGTTTTCGCGCTAAGGCTCATGCTACACTAGAGC 3'	hairpin with 5' phosphate
14	5' GCTCTAGTGTAGCATGAGCCTTAGCGCGTTTTCGCGCTAAGGCTCATGCTACACTAGAGCTCGA 3'	hairpin
15	5' GCTCTAGTGTAGCATGAGCCTTAGCGCGTTTTCGCGCTAAGGCTCATGCTACACTAGAGCTCGA 3'	hairpin with 5' phosphate
16	5' AGCTGCTCTAGTGTAGCATGAGCCTTAGCGCGTTTTCGCGCTAAGGCTCATGCTACACTAGAGC 3'	hairpin

**17**	5' AGCTGCTCTAGTGTAGCATGAGCCTTAGCGCGTTTTCGCGCTAAGGCTCATGCTACACTAGAGC 3'	hairpin with 5' phosphate
**18**	5' GGAAGTTCTTTTGAACTTCCGCGAAGCTTTTGCTTCGC 3'	dumbell
**19**	5' GGAAGTTCTTTTGAACTTCCGCGAAGCTTTTGCTTCGC 3'	dumbell with 5' phosphate
**20**	5' CAGCAGGTACCTGCCCGGCGCCCAGAGAGGAATGCAACACTC 3'	with 5' FAM fluorescein
	3' GTCGTCCATGGACGGGCCGCGGGTCTCTCCTTACGTTGTGAG 5'	
**21**	5' CAGCAGGTACCTGCCCGGCGCCCAGAGAGGAATGCAACACTC 3'	with 5' FAM fluorescein
**22**	5' GCTCTAGTGTAGCATGAGCCTTAGCGCGTTTTCGCGC**U**AAGGCTCATGCTACACTAGAGCTCGA 3'	hairpin with internal fluorescein (bolded)
**23**	5' GCTCTAGTGTAGCATGAGCCTTAGCGCGTTTTCGCGC**U**AAGGCTCATGCTACACTAGAGCTCGA 3'	hairpin with internal FAM fluorescein (bolded) and 5' phosphate

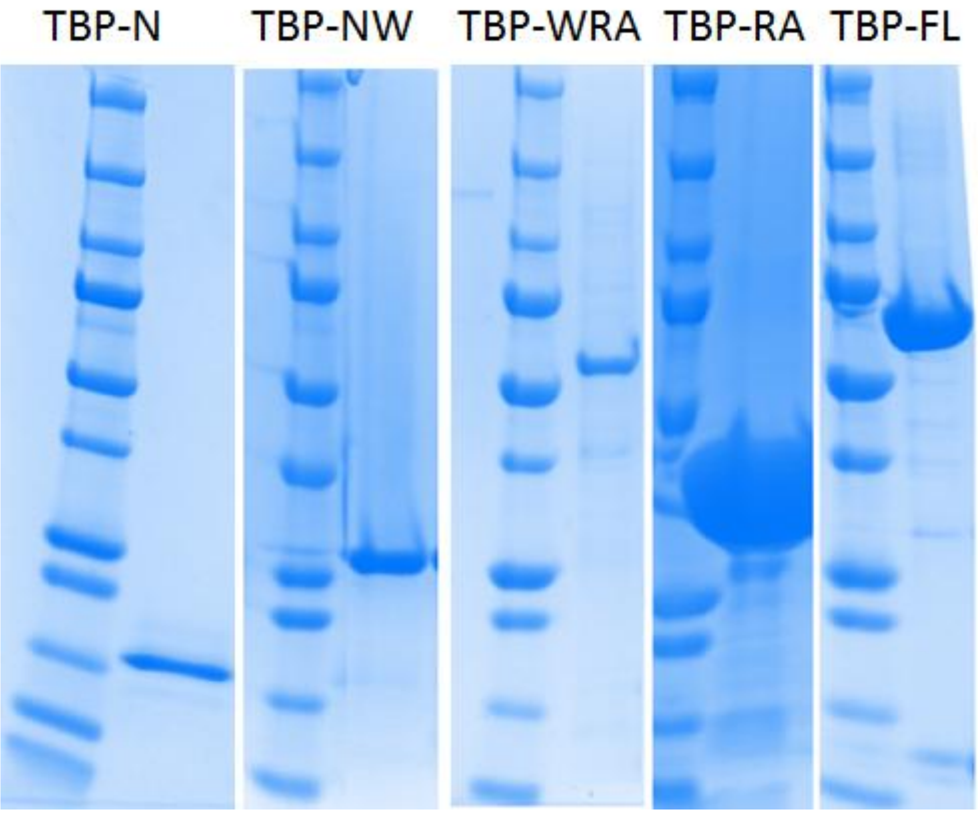

Supplementary Figure S1. Purity of the protein constructs. The proteins were resolved by SDS-PAGE using 4-15% gradient gels (Bio-Rad) and stained with PageBlue (ThermoFisher Scientific).

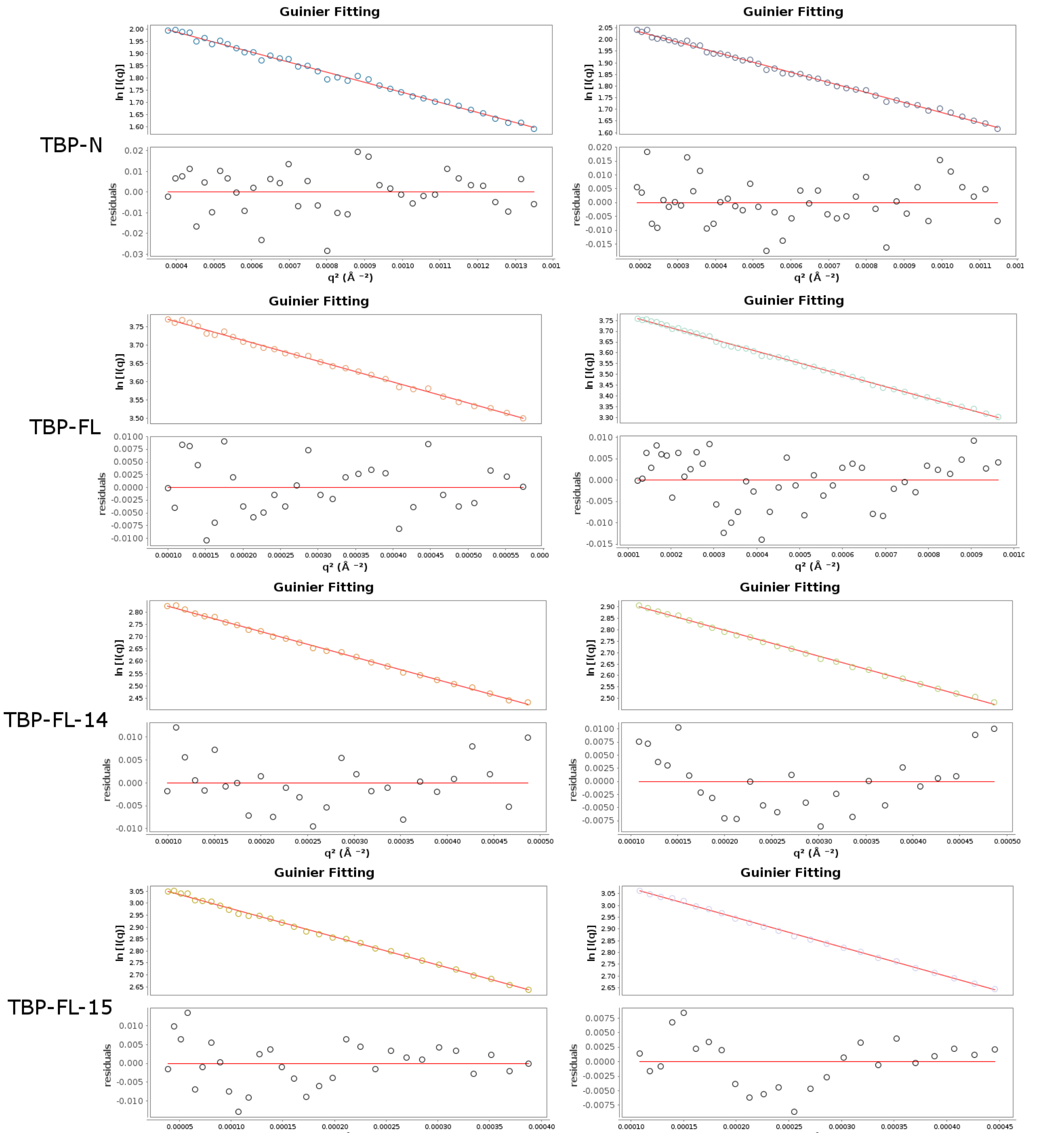

Supplementary Figure S2. Guinier plots of the SAXS data sets. The figure shows the Guinier plots for the SAXS data sets used in data analysis. The Guinier plots for lower protein concentrations (Table 1) are shown in left panels and for higher protein concentrations in right panels. The data were plotted with ScÅtter (www.bioisis.net).
